# Effect of Engineered Nanoparticles on Exopolymeric Substances Release from Marine Phytoplankton

**DOI:** 10.1186/s11671-017-2397-x

**Published:** 2017-12-13

**Authors:** Meng-Hsuen Chiu, Zafir A. Khan, Santiago G. Garcia, Andre D. Le, Agnes Kagiri, Javier Ramos, Shih-Ming Tsai, Hunter W. Drobenaire, Peter H. Santschi, Antonietta Quigg, Wei-Chun Chin

**Affiliations:** 1 Bioengineering Program, School of Engineering, University of California at Merced, Merced, CA 95343 USA; 2 Department of Marine Science, Texas A&M University Galveston campus, Galveston, TX USA; 3 Department of Marine Biology, Texas A&M University Galveston campus, Galveston, TX USA

**Keywords:** Nanoparticles, Phytoplankton, Ca^2+^ signal, Extracellular polymeric substances

## Abstract

Engineered nanoparticles (ENPs), products from modern nanotechnologies, can potentially impact the marine environment to pose serious threats to marine ecosystems. However, the cellular responses of marine phytoplankton to ENPs are still not well established. Here, we investigate four different diatom species (*Odontella mobiliensis*, *Skeletonema grethae*, *Phaeodactylum tricornutum*, *Thalassiosira pseudonana*) and one green algae (*Dunaliella tertiolecta*) for their extracellular polymeric substances (EPS) release under model ENP treatments: 25 nm titanium dioxide (TiO_2_), 10–20 nm silicon dioxide (SiO_2_), and 15–30 nm cerium dioxide (CeO_2_). We found SiO_2_ ENPs can significantly stimulate EPS release from these algae (200–800%), while TiO_2_ ENP exposure induced the lowest release. Furthermore, the increase of intracellular Ca^2+^ concentration can be triggered by ENPs, suggesting that the EPS release process is mediated through Ca^2+^ signal pathways. With better understanding of the cellular mechanism mediated ENP-induced EPS release, potential preventative and safety measures can be developed to mitigate negative impact on the marine ecosystem.

**Electronic supplementary material:**

The online version of this article (10.1186/s11671-017-2397-x) contains supplementary material, which is available to authorized users.

## Background

Engineered nanoparticles (ENPs), which range in size between 1 and 100 nm (in at least one dimension), are used in the fabrication of numerous consumer goods, including printer inks and paints, detergents, bactericides, coatings, cosmetics, sunscreen lotions, tires, computer construction, and drug delivery. Given the promising application of ENPs, funding for the National Nanotechnology Initiative (NNI) in the USA alone approached $1.4 billion in 2017 [1–3]. Establishing foundational knowledge at the nanoscale was the main focus of the nanotechnology research community in the first phase. As of 2009, this new knowledge underpinned about a quarter of a trillion dollars worldwide market, of which about $91 billion was in US products that incorporate nanoscale components [4]. With the rapid development of nanotechnology, it is inevitable that ENPs will eventually find their way to aquatic systems. The major concern with ENPs in terms of their potential toxicity (e.g., the potential for producing reactive oxygen species, ROS) in the environment is related to their large and unique surface reactivity. However, the actual impact on the marine ecosystem remains largely unknown due to complex environmental and biological factors of natural waters and variety of ENPs [1, 5, 6]. Previous studies have shown that ENPs can cause significant harm to the algae-based marine ecosystem [7, 8]. Marine organisms (particularly phytoplankton) have shown to interact with ENPs leading to negative repercussions [9–11]. With the potential increased nanotechnology utilization in diverse fields, more and more ENPs may enter aquatic environments, so the cellular responses of marine phytoplankton to ENPs warrant further attention [12–21].

Most marine microbes, whether auto- or heterotrophic, are generally capable of producing exopolymeric substances (EPS), which have diverse functional roles and physical properties in the marine ecosystem acting as growth inhibitors, growth promoters, toxins, metal scavengers, or as substrates for the heterotrophic cycle [22–26]. EPS released from phytoplankton and bacteria in the ocean are polysaccharide-rich anionic colloidal biopolymers that are critical for the formation of marine gels, marine snow, and biofilms, as well as for colloid and trace element scavenging and for providing protection against various environmental threat, including ENPs [7, 15, 19, 20, 25, 27]. In addition, the secretion of EPS is believed to be a natural response when phytoplankton experience various stress [8].

Ca^2+^ is a common second messenger involved in a multitude of intracellular signaling pathways. It has been demonstrated that Ca^2+^ is required for chemotaxis, motility, and adhesion in the diatom *Amphora coffeaeformis* [28]. Enhanced intracellular free Ca^2+^ levels are known to lead to the activation of protein kinase C, which is involved in many intracellular signaling pathways [29]. Since the release of EPS is closely related to the motility and adhesion of diatoms, it was proposed that a Ca^2+^-mediated secretion process controls the release of EPS from diatoms [30], and the direct evidence verifying Ca^2+^ signaling, exocytosis, and correlating Ca^2+^ signaling with exocytosis has been reported in our previous study [31]. Past studies have also demonstrated that interactions with ENPs can alter the intracellular Ca^2+^ pathways, which are essential for cell signaling [29, 32–34]. Specific intracellular Ca^2+^ concentration changes are important in cell signaling and secretion processes; however, there are no reports of titanium dioxide (TiO_2_), silicon dioxide (SiO_2_), or cerium dioxide (CeO_2_) to alter intracellular Ca^2+^ level in phytoplankton.

In 2013, Quigg et al. [8] summarized the direct and indirect toxic effects of ENPs on algae. In our previous experiments, ENPs were shown to facilitate EPS aggregation [35]. In this regard, EPS may either exacerbate or reduce direct ENP-induced toxicity toward aquatic organisms [7, 15, 36]. However, direct measurement for EPS release from phytoplankton under ENPs stress has never been reported. In this study, the aim is to study the release of EPS from four different diatom species (*Odontella mobiliensis*, *Skeletonema grethae*, *Phaeodactylum tricornutum*, *Thalassiosira pseudonana*) and one green algae (*Dunaliella tertiolecta*) under ENP treatments. By understanding underlying mechanisms of ENP-induced EPS-release in phytoplankton, implementation of preventative and safety measures can mitigate potentially detrimental effects toward marine organisms.

## Results and Discussions

### ENP Characterization

**Fig. 1:**
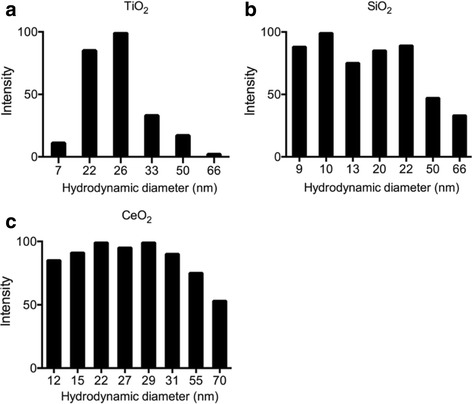
ENP characterization by DLS assessment of **a** TiO_2_, **b** SiO_2_, and **c** CeO_2_ in L1 medium after sonication showing their size distribution. The ENP final concentration in DLS sample is 1 μg/ml, the measuring time is 3 min right after the sonication

Dynamic laser scattering (DLS) was used to characterize size metrics of the following ENPs suspended in pure water: TiO_2_, SiO_2_, and CeO_2_. The particle size distribution ranged from 7 to 66 nm in TiO_2_, 9 to 66 nm in SiO_2_, and 12 to 70 nm in CeO_2_. Some larger sizes could be due to aggregation or agglomeration while the predominant size for TiO_2_ is 25 nm, SiO_2_ is 10 to 20 nm, and CeO_2_ is 15 to 30 nm, which are consistent with manufacturer’s information (Fig. 1).

### ENPs Induce Intracellular Ca^2+^ Concentration in Phytoplankton

**Fig. 2:**
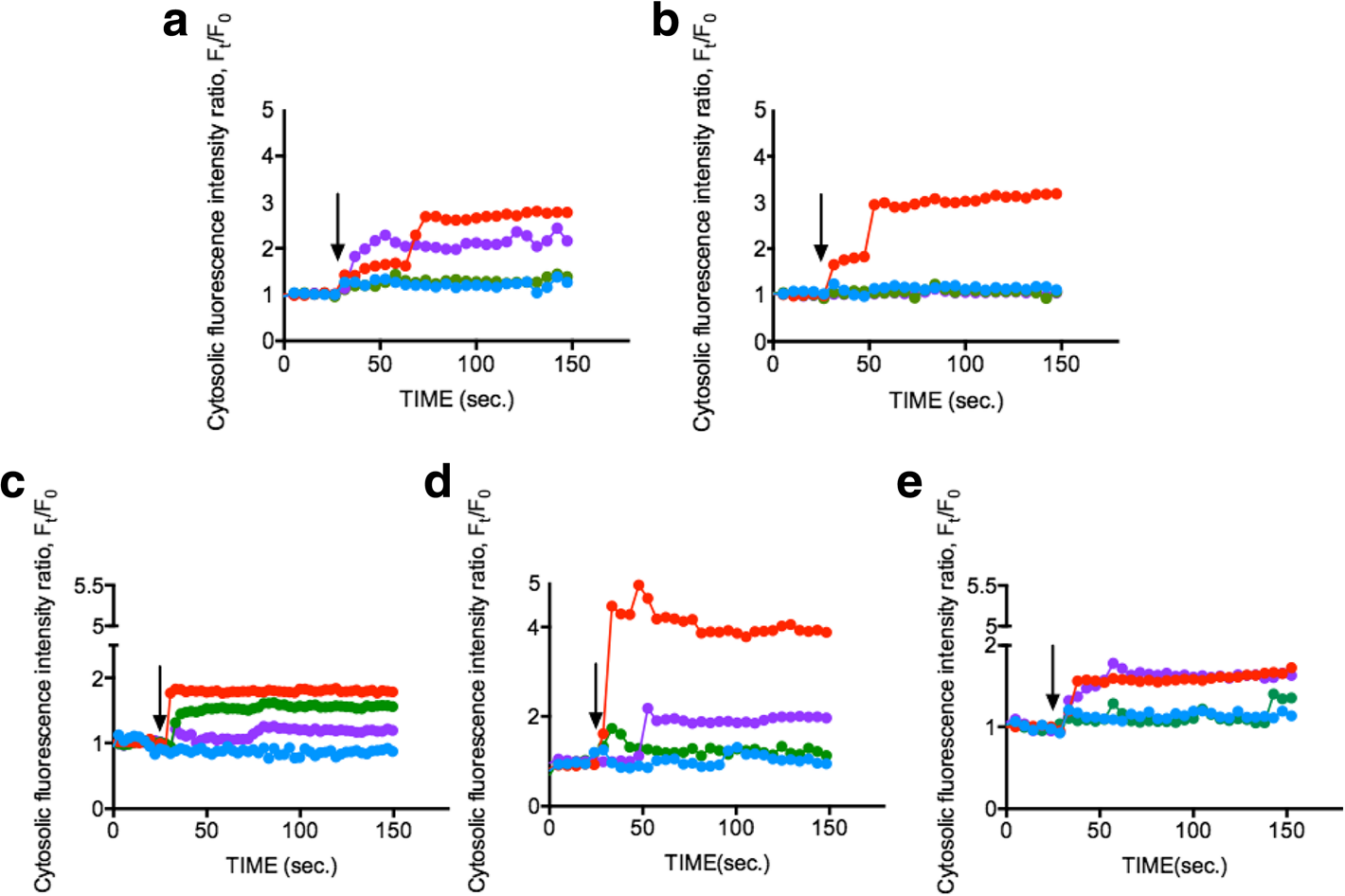
Measurement of intracellular Ca^2+^ concentration after stimulation by different ENPs. Different phytoplankton cells **a** *Dunaliella tertiolecta*, **b** *Thalassiosira pseudonana*, **c** *Skeletonema grathae*, **d** *Phaeodactylum tricornutum*, and **e** *Odontella mobiliensis* were treated with TiO_2_ 25 nm (green), SiO_2_ 10–20 nm (red), CeO_2_ 15–30 nm (purple) with a concentration of 1 mg/ml and control (blue). Black arrow indicates the time point when EPNs were applied (30 s). The measurements show representative data from an average of 20 individual cells

To investigate whether ENPs could induce an increase in intracellular Ca^2+^ concentration, phytoplankton cells (OD 600 = 0.8) were loaded with Fluo-4AM dye and exposed to 1 mg/ml of 25 nm TiO_2_, 10–20 nm SiO_2_, and 15–30 nm CeO_2_ ENPs respectively. The change in intracellular Ca^2+^ concentration, as represented by the fluorescence intensity within phytoplankton cells, was monitored for 150 s. Figure 2a–e show that 1 mg/ml of three respective ENPs increased Ca^2+^ concentration in SiO_2_ by approximately 50–300%, TiO_2_ by approximately 40%, and CeO_2_ by approximately 150–200%, while the control conditions (L1 medium) remained unchanged. The results show ENPs can induced significant intracellular Ca^2+^ responses in phytoplankton and suggest that phytoplankton respond to distinct ENPs through Ca^2+^ signaling pathways. Our data indicates only minor changes in intracellular Ca^2+^ levels when TiO_2_ is present, potentially attributed to substantial phytoplankton cell death from TiO_2_-induced toxicity [37, 38]. In our previous study, TiO_2_ prompted increase in the intracellular Ca^2+^ concentration [34] alongside significant cell apoptosis [39]. However, SiO_2_ surprisingly showed the most obvious intracellular Ca^2+^ increase for all phytoplankton species, while CeO_2_ can only trigger an intermediate intracellular Ca^2+^ concentration increase. Previous research suggested potential of high CeO_2_ concentrations (> 50 mg/ml) to induce intracellular oxidative stress and elevation of intracellular Ca^2+^ levels, though effects were small, and supported our finding [40]. We also measured the zeta potential of each ENPs in artificial seawater to address the potential effect may cause by the surface charge; however, the value was low. The measurement indicated the ENPs are considered approximately neutral [41] (Additional file 1: Supplement data). This served as the first report wherein disparate ENPs were found to induce intracellular Ca^2+^ concentration changes in specific phytoplankton, ultimately paving a new avenue for future research.

### ENP-Induced EPS Release in Phytoplankton

**Fig. 3:**
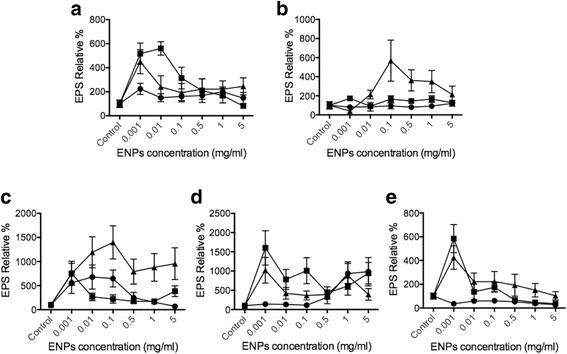
EPS release triggered by various ENPs. Different phytoplankton cells **a** *Dunaliella tertiolecta*, **b** *Thalassiosira pseudonana*, **c** *Skeletonema grathae*, **d** *Phaeodactylum tricornutum*, and **e** *Odontella mobiliensis* were treated with TiO_2_ (circles), SiO_2_ (triangles), CeO_2_ (squares), respectively, with concentrations of 5 mg/ml and 1 mg/ml, 0.5 mg/ml, 0.1 mg/ml, 10 μg/ml, 1 μg/ml (*n* = 3)

Enzyme-linked lectin assay (ELLA) was used to assess the amount of EPS release from phytoplankton cells when stimulated with TiO_2_, SiO_2_, and CeO_2_ ENPs, concentration range from 1 μg/ml to 5 mg/ml based on previous studies for TiO_2_ [42, 43] and CeO_2_ [44–46]. EPS secretion was normalized to total phytoplankton DNA amount (Additional file 1: Supplement data) in order to have an equal base for comparison. Compared with the control, we found that 10–20 nm SiO_2_ is able to increase EPS release by up to 550% in *Dunaliella*, 500% in *Thalassiosira*, 1000% in *Skeletonema*, 400% in *Odontella*, and 900% in *Phaeodactylum* (Fig. 3). When the phytoplankton species were exposed to TiO_2_, there was no strong effect on EPS secretion, as only *Skeletonema* and *Phaeodactylum* showed significant changes. EPS release data are thus consistent with our intracellular Ca^2+^ concentration results. TiO_2_ did not present a significant impact on the production of EPS, similar to the fact that intracellular Ca^+2^ concentrations showed very limit changes due to the toxicity of TiO_2_ to phytoplankton. The production and residues of ROS can lead to many complications such as apoptosis in the phytoplankton [47–49]. In the CeO_2_ treatment, results showed minor effect in *Dunaliella*, *Skeletonema*, *Odontella*, and *Phaeodactylum*. However, SiO_2_ showed the most significant EPS induction in *Thalassiosira pseudonana* (around 600%) and *Skeletonema grethae* (around 1000–1500%). These data indicate that different ENPs can induce specific EPS release from phytoplankton, and intracellular Ca^2+^ changes also match EPS release results. By assessing the changes in intracellular Ca^2+^ concentration, it is evident that there is a direct connection in the Ca^2+^ cellular pathways in which ENPs evoke the EPS secretion from phytoplankton. The observation here is in agreement with our previous studies based on *Phaeocystis* EPS release [31]. The results provide direct evidence that phytoplankton can detect and distinguish ENPs responding with different EPS release regulated by Ca^2+^ cellular pathways.

The use of ELLA allowed us to determine the release of EPS via the interactions of the phytoplankton with the ENPs. Our results indicate that EPS secretion was significantly increased as the phytoplankton interacted with SiO_2_ for *Dunaliella tertiolecta*, *Thalassiosira pseudonana*, and *Skeletonema grethae*. It appears that these diatoms are primed to recognize SiO_2_ particles. However, in *Phaeodactylum tricornutum*, a strong EPS secretion was not found. This difference represents EPS release triggered by ENPs dependents on the phytoplankton species and ENPs concentration (Fig. 3). In a previous study, oil spills caused large marine microbial EPS releases that were proposed to counteract the negative consequence of oil spills [50]. In addition, Boglaienko, and Tansel found that SiO_2_ particles was able to remove oil aggregates efficiently [51]. Our finding provides a new potential mechanism wherein low toxicity SiO_2_ particles can induce EPS release from specific phytoplankton, potentially facilitating oil-spill removal by promoting EPS aggregation. Cerium dioxide has never been reported to disturb phytoplankton-based marine ecosystems. Results here showed CeO_2_ ENPs can impact all phytoplankton here except *Thalassiosira pseudonana.* CeO_2_ ENPs may, like SiO_2,_ have the ability to boost EPS release from particular phytoplankton for oil mitigation applications.

## Conclusions

The ENP-marine environment interaction is becoming increasingly critical due to current and future discharges of nanomaterials. Here, we demonstrate enhanced EPS secretion as one of the major effects of ENPs to phytoplankton. We also provide evidence that different phytoplankton can respond differently to various ENP stresses by regulating Ca^2+^ pathways. However, a complete assessment of ENPs to marine ecosystem would need further investigations to provide detailed knowledge and understanding of the interactions between nanomaterials and marine organisms.

## Methods

### Phytoplankton Culture

Batch cultures of *Odontella mobiliensis* (CCMP597), *Dunaliella tertiolecta* (UTEX999), *Skeletonema grethae* (CCMP775), *Phaeodactylum tricornutum* (UTEX646), *Thalassiosira pseudonana* (Provasoli - Guillard marine phytoplankton culture collection, West Boothbay Harbor, MN, USA) were grown in L1 marine medium (Sigma, MO, USA) on a 14:10 (light: dark) cycle at 100 μmol m^−2^ s^−1^ and 24 °C under axenic conditions. Growth phase of the culture was determined by cell counting with a hemocytometer.

### Nanoparticles and Characterization

All ENPs, TiO_2_, SiO_2_, CeO_2_ (Sigma-Aldrich, MO, USA), were sonicated in pure water before usage. ENPs were reconstituted with filtered L1 medium (Sigma, MO, USA) before being tested. The size of ENPs was independently confirmed using homodyne dynamics laser scattering (DLS). Briefly, seawater samples were refiltered through a 0.22-μm Millipore membrane (pre-washed with 0.1N HCl) and poured directly into five 10 ml scattering cells that were then positioned in the goniometer of a Brookhaven BI-200SM laser spectrometer (Brookhaven Instruments, NY, USA). The autocorrelation function of the scattering intensity fluctuations detected at a 45° angle was processed on line by a Brookhaven BI 9000ATautocorrelator, and particle size distribution was calculated by the CONTIN method (Provencher, 1982). Results from each sample were collected in triplicate right after sonication. Calibration of the DLS spectrometer was conducted using standard suspensions of monodisperse latex microspheres (Polysciences, PA, USA).

### ENP Treatment

The phytoplankton cells were cultured in a 96-well plate with L1 medium for 24 h. Cells were treated with ENP stocks: 5 mg/ml and 1 mg/ml, 0.5 mg/ml, 0.1 mg/ml, 10 μg/ml, 1 μg/ml of the TiO_2_, SiO_2_, and CeO_2_ (Sigma-Aldrich, MO, USA) or L1 medium (control) for 48 h. The supernatant containing secreted EPS was collected and briefly centrifuged at 4000 rpm to remove the residual ENPs. This protocol was adapted from our previous publication [34]. The concentration range used here is not intended to represent or mimic the current ENP levels in the environment but aims to assess the full potential impact of ENPs on marine phytoplankton and investigate the associate cellular mechanisms. As a promising emergent nanomaterial, ENPs have not yet reached their full commercial capacity. Detailed assessment of their complete ecological impacts is much needed before ENPs enter commercial and household product market to introduce more ENPs into the ocean.

### Enzyme-Linked Lectin Assay (ELLA)

The supernatant containing secreted polysaccharide was collected and briefly centrifuged at 1700 rcf (Megafuge 1.0R) to remove the residual ENPs. The supernatant was then incubated in a 96 well (Nunc MaxiSorp, VWR, CA, USA) plate overnight at 4 °C. Afterwards the 96-well plate was washed with PBST (PBS + 0.05% Tween-20) and PBS and then blocked with 1% BSA. The 96-well plate was washed again with PBST and PBS and incubated with lectin (Concanavalin A, ConA) (Sigma-Aldrich, MO, USA), conjugated to horseradish peroxidase (HRP; 5 mg/ml) (Sigma-Aldrich, MO, USA), at 37 °C for 1 h. The substrate, 3,39,5,59-tetramethylbenzidine (TMB; Sigma-Aldrich, MO, USA), was added to each well at room temperature followed by H_2_SO4 (Sigma-Aldrich, MO, USA) in order to terminate the reaction. The optical density was measured at 450 nm by PerkinElmer VICTOR3 (MA, USA). This protocol was adapted from our previous publication [34, 52].

### DNA Determination

The pellet containing phytoplankton was collected and obtained the ZR-96 Quick-gDNA kit (ZYMO Research, CA, USA). In brief, 4× lysis buffer was used to break phytoplankton cells and flow through the DNA binding column, eluted by elution buffer in the end. DNA concentrations were measured by NanoDrop ND-1000 (Thermo, CA, USA). Protocol was adapted from manufactured kit protocol.

### Measurements of Intracellular Ca^2+^ Concentrations Induced by ENPs

The phytoplankton cells were then loaded with a Fluo-4AM dye (1 mM) (Kd = 335 nM, λEx = 494 nm, and λEm = 506 nm, ThermoFisher, CA, USA) for 60 min [31]. After the dye loading, the phytoplankton cells were rinsed, incubated with L1 medium, and treated with the 1 mg/ml TiO_2_, SiO_2_, and CeO_2_ respectively. All calcium signaling experiments were carried out on a Nikon microscope (Nikon Eclipse TE2000-U, Tokyo, Japan). Protocol and conditions were adapted from previous publications [31, 34].

### Zeta Potential of ENP Measurement

To measure the surface charges of ENPs, the zeta potential (ζ) of ENPs was measured with a Zetasizer Nano ZS, Malvern, in the presence of artificial seawater at 25 °C. After the data were collected from each sample, the recorded values were averaged.

### Statistical Analysis

The data is reported as means ± SD. Each experiment was performed independently at least three times. Histograms were made by GraphPad Prism 6.0. (GraphPad Software, Inc., San Diego, CA, USA).

## Additional file

Additional file 1: Supplement data. (DOCX 224 kb)

## References

[1] Guzman KAD, Taylor MR, Banfield JF (2006). Environmental risks of nanotechnology: national nanotechnology initiative funding, 2000–2004. Environmental science & technology.

[2] Roco MC (2005). International perspective on government nanotechnology funding in 2005. J Nanopart Res.

[3] Service RF (2004). Nanotoxicology: nanotechnology grows up. Science.

[4] Roco MC (2011). The long view of nanotechnology development: the National Nanotechnology Initiative at 10 years. J Nanopart Res.

[5] Fabrega J, Luoma SN, Tyler CR, Galloway TS, Lead JR (2011). Silver nanoparticles: behaviour and effects in the aquatic environment. Environ Int.

[6] Oberdorster G, Oberdorster E, Oberdorster J (2005). Nanotoxicology: an emerging discipline evolving from studies of ultrafine particles. Environ Health Perspect.

[7] Miao AJ, Zhang XY, Luo Z, Chen CS, Chin WC, Santschi PH, Quigg A (2010). Zinc oxide-engineered nanoparticles: dissolution and toxicity to marine phytoplankton. Environ Toxicol Chem.

[8] Quigg A, Chin W-C, Chen C-S, Zhang S, Jiang Y, Miao A-J, Schwehr KA, Xu C, Santschi PH (2013). Direct and indirect toxic effects of engineered nanoparticles on algae: role of natural organic matter. ACS Sustain Chem Eng.

[9] Kadar E, Rooks P, Lakey C, White DA (2012). The effect of engineered iron nanoparticles on growth and metabolic status of marine microalgae cultures. Sci Total Environ.

[10] Navarro E, Baun A, Behra R, Hartmann NB, Filser J, Miao AJ, Quigg A, Santschi PH, Sigg L (2008). Environmental behavior and ecotoxicity of engineered nanoparticles to algae, plants, and fungi. Ecotoxicology.

[11] Jarvis TA, Miller RJ, Lenihan HS, Bielmyer GK (2013). Toxicity of ZnO nanoparticles to the copepod Acartia tonsa, exposed through a phytoplankton diet. Environ Toxicol Chem.

[12] Dubois F, Mahler B, Dubertret B, Doris E, Mioskowski C (2007). A versatile strategy for quantum dot ligand exchange. J Am Chem Soc.

[13] Mao JA, Bai Y, Gu L, van Aken PA, Tu MJ (2010). Preparation and characterization of size-controlled CeO2 nanoparticles coated with SiO2. J Nanopart Res.

[14] Miao AJ, Luo ZP, Chen CS, Chin WC, Santschi PH, Quigg A (2010) Intracellular uptake: a possible mechanism for silver engineered nanoparticle toxicity to a freshwater alga Ochromonas danica. PLoS One 5(12) 10.1371/journal.pone.0015196 PMC3008680 21203552

[15] Miao AJ, Schwehr KA, Xu C, Zhang SJ, Luo ZP, Quigg A, Santschi PH (2009). The algal toxicity of silver engineered nanoparticles and detoxification by exopolymeric substances. Environ Pollut.

[16] Pellegrino T, Manna L, Kudera S, Liedl T, Koktysh D, Rogach AL, Keller S, Radler J, Natile G, Parak WJ (2004). Hydrophobic nanocrystals coated with an amphiphilic polymer shell: a general route to water soluble nanocrystals. Nano Lett.

[17] Wang Y, Wong JF, Teng XW, Lin XZ, Yang H (2003). “Pulling” nanoparticles into water: phase transfer of oleic acid stabilized monodisperse nanoparticles into aqueous solutions of alpha-cyclodextrin. Nano Lett.

[18] Yu WW, Chang E, Falkner JC, Zhang JY, Al-Somali AM, Sayes CM, Johns J, Drezek R, Colvin VL (2007). Forming biocompatible and nonaggregated nanocrystals in water using amphiphilic polymers. J Am Chem Soc.

[19] Zhang SJ, Jiang YL, Chen CS, Creeley D, Schwehr KA, Quigg A, Chin WC, Santschi PH (2013). Ameliorating effects of extracellular polymeric substances excreted by Thalassiosira pseudonana on algal toxicity of CdSe quantum dots. Aquat Toxicol.

[20] Zhang SJ, Jiang YL, Chen CS, Spurgin J, Schwehr KA, Quigg A, Chin WC, Santschi PH (2012). Aggregation, dissolution, and stability of quantum dots in marine environments: importance of extracellular polymeric substances. Environ Sci Technol.

[21] Zhang TR, Ge JP, Hu YX, Yin YD (2007). A general approach for transferring hydrophobic nanocrystals into water. Nano Lett.

[22] Liu MS, Hellebust JA (1974). Uptake of amino acids by the marine centric diatom Cyclotella cryptica. Can J Microbiol.

[23] Xue Z, Sendamangalam VR, Gruden CL, Seo Y (2012). Multiple roles of extracellular polymeric substances on resistance of biofilm and detached clusters. Environ Sci Technol.

[24] Bhaskar P, Bhosle NB (2005). Microbial extracellular polymeric substances in marine biogeochemical processes.

[25] Hoagland KD, Rosowski JR, Gretz MR, Roemer SC (1993). Diatom extracellular polymeric substances: function, fine structure, chemistry, and physiology. J Phycol.

[26] Passow U, Alldredge AL (1995). Aggregation of a diatom bloom in a mesocosm: the role of transparent exopolymer particles (TEP). Deep-Sea Res II Top Stud Oceanogr.

[27] Decho AW, Herndl GJ (1995). Microbial activities and the transformation of organic-matter within mucilaginous material. Sci Total Environ.

[28] Cooksey KE (1981). Requirement for calcium in adhesion of a fouling diatom to glass. Appl Environ Microbiol.

[29] Scherbart AM, Langer J, Bushmelev A, van Berlo D, Haberzettl P, van Schooten F-J, Schmidt AM, Rose CR, Schins RP, Albrecht C (2011). Contrasting macrophage activation by fine and ultrafine titanium dioxide particles is associated with different uptake mechanisms. Particle and fibre toxicology.

[30] Cooksey K, Wigglesworth-Cooksey B (1995). Adhesion of bacteria and diatoms to surfaces in the sea: a review. Aquat Microb Ecol.

[31] Chin WC, Orellana MV, Quesada I, Verdugo P (2004). Secretion in unicellular marine phytoplankton: demonstration of regulated exocytosis in Phaeocystis globosa. Plant Cell Physiol.

[32] Wang H-J, Growcock AC, Tang T-H, O’Hara J, Huang Y-W, Aronstam RS (2010). Zinc oxide nanoparticle disruption of store-operated calcium entry in a muscarinic receptor signaling pathway. Toxicol in Vitro.

[33] Horie M, Nishio K, Kato H, Fujita K, Endoh S, Nakamura A, Miyauchi A, Kinugasa S, Yamamoto K, Niki E (2011). Cellular responses induced by cerium oxide nanoparticles: induction of intracellular calcium level and oxidative stress on culture cells. J Biochem.

[34] Chen EY, Garnica M, Wang YC, Chen CS, Chin WC (2011). Mucin secretion induced by titanium dioxide nanoparticles. PLoS One.

[35] Miao A-J, Luo Z, Chen C-S, Chin W-C, Santschi PH, Quigg A (2010). Intracellular uptake: a possible mechanism for silver engineered nanoparticle toxicity to a freshwater alga Ochromonas danica. PLoS One.

[36] Chen C-S, Anaya JM, Zhang S, Spurgin J, Chuang C-Y, Xu C, Miao A-J, Chen EY, Schwehr KA, Jiang Y (2011). Effects of engineered nanoparticles on the assembly of exopolymeric substances from phytoplankton. PLoS One.

[37] Manzo S, Buono S, Rametta G, Miglietta M, Schiavo S, Di Francia G (2015). The diverse toxic effect of SiO2 and TiO2 nanoparticles toward the marine microalgae Dunaliella tertiolecta. Environ Sci Pollut R.

[38] Schiavo S, Oliviero M, Miglietta M, Rametta G, Manzo S (2016). Genotoxic and cytotoxic effects of ZnO nanoparticles for Dunaliella tertiolecta and comparison with SiO2 and TiO2 effects at population growth inhibition levels. Sci Total Environ.

[39] Chen E, Ruvalcaba M, Araujo L, Chapman R, Chin W-C (2008). Ultrafine titanium dioxide nanoparticles induce cell death in human bronchial epithelial cells. J Exp Nanosci.

[40] Horie M, Nishio K, Kato H, Fujita K, Endoh S, Nakamura A, Miyauchi A, Kinugasa S, Yamamoto K, Niki E, Yoshida Y, Hagihara Y, Iwahashi H (2011). Cellular responses induced by cerium oxide nanoparticles: induction of intracellular calcium level and oxidative stress on culture cells. J Biochem.

[41] Clogston JD, Patri AK (2011). Zeta potential measurement.

[42] Theogaraj E, Riley S, Hughes L, Maier M, Kirkland D (2007). An investigation of the photo-clastogenic potential of ultrafine titanium dioxide particles. Mutation Research/Genetic Toxicology and Environmental Mutagenesis.

[43] Falck G, Lindberg H, Suhonen S, Vippola M, Vanhala E, Catalan J, Savolainen K, Norppa H (2009). Genotoxic effects of nanosized and fine TiO2. Human & experimental toxicology.

[44] Cassee FR, van Balen EC, Singh C, Green D, Muijser H, Weinstein J, Dreher K (2011). Exposure, health and ecological effects review of engineered nanoscale cerium and cerium oxide associated with its use as a fuel additive. Crit Rev Toxicol.

[45] Das S, Dowding JM, Klump KE, McGinnis JF, Self W, Seal S (2013). Cerium oxide nanoparticles: applications and prospects in nanomedicine. Nanomedicine.

[46] Van Hoecke K, Quik JT, Mankiewicz-Boczek J, De Schamphelaere KA, Elsaesser A, Van der Meeren P, Barnes C, McKerr G, Howard CV, Van de Meent D, Rydzynski K, Dawson KA, Salvati A, Lesniak A, Lynch I, Silversmit G, De Samber B, Vincze L, Janssen CR (2009). Fate and effects of CeO2 nanoparticles in aquatic ecotoxicity tests. Environmental science & technology.

[47] Aguirre J, Rios-Momberg M, Hewitt D, Hansberg W (2005). Reactive oxygen species and development in microbial eukaryotes. Trends Microbiol.

[48] Vardi A, Formiggini F, Casotti R, De Martino A, Ribalet F, Miralto A, Bowler C (2006). A stress surveillance system based on calcium and nitric oxide in marine diatoms. PLoS Biol.

[49] Faria M, Navas JM, Soares AM, Barata C (2014). Oxidative stress effects of titanium dioxide nanoparticle aggregates in zebrafish embryos. Sci Total Environ.

[50] Gutierrez T, Berry D, Yang T, Mishamandani S, McKay L, Teske A, Aitken MD (2013). Role of bacterial exopolysaccharides (EPS) in the fate of the oil released during the deepwater horizon oil spill. PLoS One.

[51] Boglaienko D, Tansel B (2015). Instantaneous stabilization of floating oils by surface application of natural granular materials (beach sand and limestone). Mar Pollut Bull.

[52] Leriche V, Sibille P, Carpentier B (2000). Use of an enzyme-linked lectinsorbent assay to monitor the shift in polysaccharide composition in bacterial biofilms. Appl Environ Microbiol.

