# Supplementary material for: Effect of Engineered Nanoparticles on Exopolymeric Substances Release from Marine Phytoplankton
Source: Nanoscale Res Lett. 2017 Dec 13;12:620. doi: 10.1186/s11671-017-2397-x (PMC5729174; doi:10.1186/s11671-017-2397-x)
Supplement: Additional file 1: — Supplement data. (DOCX 224 kb) [file 11671_2017_2397_MOESM1_ESM.docx]

Supplement Data

Figure. S1

A

**

B

**

C

D

E

**DNA amount of individual phytoplankton at varying concentrations of ENPs.**

Five phytoplankton isolates treat with three different ENPs at varying concentrations and after 48 hrs, the DNA was collected by DNA extraction kit and quantified by NanoDrop.

Table. S1

| **ENPs** | **Zeta Potential [mV]** |
| --- | --- |
| TiO_2_ | 7.8±1.1 |
| CeO_2_ | 8.8±1.4 |
| SiO_2_ | -0.33±3.2 |

**The zeta potential of ENPs in ASW.**

Equilibrium size ENPs at ASW.
